# Full-time Work Rates of Physicians With Physician Spouses vs Nonphysician Spouses in Japan

**DOI:** 10.1001/jamanetworkopen.2022.42143

**Published:** 2022-11-14

**Authors:** Atsushi Miyawaki

**Affiliations:** 1 Department of Public Health, Graduate School of Medicine, The University of Tokyo, Tokyo, Japan

## Abstract

This cross-sectional study investigates the association of physician vs nonphysician spousal occupation with full-time work rates among married physicians.

## Introduction

With an increasing number of female physicians, dual-physician marriages (ie, physicians married to physicians) are increasingly a topic of discourse in the medical literature.^1,2^ However, there are limited national-level data on the association of spousal occupation with physician labor force participation.^3,4^

## Methods

The University of Tokyo ethics committee approved this cross-sectional study and granted a waiver of informed consent because we used deidentified data. The STROBE reporting guideline was followed. We used stratified random samples from the Japan Population Census (2005-2015), a quinquennial survey conducted on October 1, collecting information on family structures, demographics, and employment of all Japanese residents (95%-100% response rates).^5^

Married individuals working as physicians were analyzed. Specialty data were unavailable. We limited analyses to couples aged 25 to 50 years (ie, child-rearing years). Same-sex couples were excluded. The outcome was a full-time work indicator, dichotomized for physicians (1 = “primarily worked,” and 0 = “worked part time besides housework,” “worked part time while studying,” or “on leave”) according to the question, “How did you work during the week of September 24-30?” (eMethods in the Supplement).

We used multivariable modified Poisson regression models separately for male and female physicians to investigate the association between spousal occupation (physician or nonphysician) and full-time work rates. We adjusted for both spouses’ ages, youngest child’s age, number of children, urbanicity of residence, prefecture, and year indicators. Analyses were repeated after (1) further subgrouping physicians married to nonphysicians into those married to individuals in other high-income occupations vs others or (2) stratifying physicians by youngest child age. High-income occupations were determined by mean income per occupation (eMethods in the Supplement). Analyses were conducted using Stata statistical software version 17 (StataCorp) from June 24 to September 21, 2022.

## Results

We observed 25 321 married physicians: 20 858 men (82.4%; mean [SD] age, 40.8 [6.2] years) and 4463 women (17.6%; mean [SD] age, 37.9 [5.8] years) (Table 1). Approximately 15% of male physicians and 69% of female physicians had a physician spouse.

**Table 1.** Characteristics of Married Physicians

Characteristic	Physicians, weighted No. (%) (N = 25 321)^a^					
	Men			Women		
	Total (n = 20 858)	Physician spouse (n = 3074)	Nonphysician spouse (n = 17 783)	Total (n = 4463)	Physician spouse (n = 3074)	Nonphysician spouse (n = 1389)
Age, mean (SD), y						
Physician	40.8 (6.2)	39.4 (6.3)	41.1 (6.1)	37.9 (5.8)	37.7 (6.0)	38.3 (5.5)
Spouse	38.1 (6.0)	37.7 (6.0)	38.1 (6.0)	39.4 (6.2)	39.4 (6.3)	39.4 (6.0)
Spouse’s occupation						
Physician	3074 (14.7)	3074 (100)	0	3074 (68.9)	3074 (100)	0
Other high-income occupation^b^	524 (2.5)	0	524 (2.9)	594 (13.3)	0	594 (42.8)
Non–high-income occupation	5328 (25.5)	0	5328 (30.0)	711 (15.9)	0	711 (51.2)
Unemployed	11 931 (57.2)	0	11 931 (67.1)	84 (1.9)	0	84 (6.0)
Age of youngest child, y^c^						
No children	3548 (17.0)	718 (23.4)	2830 (15.9)	1119 (25.1)	718 (23.4)	401 (28.9)
0-3	6473 (31.0)	990 (32.2)	5483 (30.8)	1467 (32.9)	990 (32.2)	477 (34.3)
4-6	3272 (15.7)	474 (15.4)	2798 (15.7)	665 (14.9)	474 (15.4)	191 (13.8)
≥7	7565 (36.3)	893 (29.0)	6672 (37.5)	1212 (27.2)	893 (29.0)	320 (23.0)
Children, No.						
0	3548 (17.0)	771 (25.1)	2830 (15.9)	1119 (25.1)	718 (23.4)	401 (28.9)
1	5482 (26.3)	854 (27.8)	4628 (26.0)	1323 (29.6)	854 (27.8)	469 (33.8)
2	8008 (38.4)	1090 (35.5)	6918 (38.9)	1510 (33.8)	1090 (35.5)	420 (30.2)
≥3	3820 (18.3)	412 (13.4)	3408 (19.2)	511 (11.5)	412 (13.4)	99 (7.1)
Living in urban area^d^	18 158 (87.1)	2780 (90.4)	15 378 (86.5)	4002 (89.7)	2780 (90.4)	1222 (88.0)
Year of census						
2005	7410 (35.5)	903 (29.4)	6507 (36.6)	1233 (27.6)	903 (29.4)	330 (23.8)
2010	7008 (33.6)	1006 (32.7)	6002 (33.8)	1500 (33.6)	1006 (32.7)	494 (35.6)
2015	6439 (30.9)	1165 (37.9)	5274 (29.7)	1730 (38.8)	1165 (37.9)	565 (40.7)

^a^ Analysis was conducted using stratified random samples of the Japan Population Census (an 11.7% sample for physicians). To provide nationally representative estimates, census-provided weights were used; therefore, numbers of physicians in each category may not add up to the total number of physicians.

^b^ Other high-income occupations were determined based on mean annual income per occupation (eMethods in the Supplement).

^c^ Ages at the end of the academic year were used to determine categories for youngest child age.

^d^ Urban areas were defined as census-defined densely inhabited districts.

Nearly all male physicians worked full time with or without a physician spouse (Table 2). In contrast, adjusted full-time work rates were lower for female physicians married to physicians vs those married to nonphysicians (68.1% vs 76.3%; *P* < .001). However, there was no significant difference in adjusted full-time work rates between female physicians married to physicians and those with spouses in other high-income occupations (68.3% vs 71.8%; *P* = .10).

**Table 2.** Adjusted Full-time Work Rates by Occupation of Spouse

Spouse’s occupation	Male physician				Female physician			
	Weighted No.	Adjusted full-time work rate (95% CI), %	Adjusted rate ratio (95% CI)^a^	*P* value	Weighted No.	Adjusted full-time work rate (95% CI), %	Adjusted rate ratio (95% CI)^a^	*P* value
**Main analysis**								
Physician	3074	97.7 (97.1-98.2)	1 [Reference]	NA	3074	68.1 (67.2-69.0)	1 [Reference]	NA
Nonphysician	17 783	97.9 (97.8-98.0)	1.00 (1.00-1.01)	.51	1389	76.3 (74.4-78.3)	1.12 (1.08-1.16)	<.001
**By income level of spouse’s occupation**								
Physician	3074	97.6 (97.0-98.2)	1 [Reference]	NA	3074	68.3 (67.3-69.3)	1 [Reference]	NA
Other high-income occupation	524	97.3 (96.1-98.5)	1.00 (0.98-1.01)	.70	594	71.8 (68.3-75.3)	1.05 (0.99-1.12)	.10
Other^b^	17 259	97.9 (97.8-98.0)	1.00 (1.00-1.01)	.44	795	78.9 (76.5-81.3)	1.15 (1.11-1.20)	<.001
**By age of youngest child, y^c^**								
No children								
Physician	718	95.7 (94.3-97.2)	1 [Reference]	NA	718	86.0 (84.5-87.6)	1 [Reference]	NA
Nonphysician	2830	95.7 (95.3-96.1)	1.00 (0.98-1.02)	.99	401	87.4 (84.7-90.1)	1.02 (0.97-1.07)	.52
0-3								
Physician	990	96.3 (94.9-97.7)	1 [Reference]	NA	990	53.9 (52.0-55.8)	1 [Reference]	NA
Nonphysician	5483	96.4 (96.2-96.7)	1.00 (0.98-1.02)	.89	477	66.1 (62.2-70.0)	1.23 (1.12-1.35)	<.001
4-6								
Physician	474	98.1 (97.2-99.1)	1 [Reference]	NA	474	68.5 (65.6-71.4)	1 [Reference]	NA
Nonphysician	2798	98.7 (98.6-98.9)	1.01 (0.99-1.02)	.28	191	80.7 (73.4-88.0)	1.18 (1.03-1.34)	.02
≥7								
Physician	893	99.5 (99.1-100.0)	1 [Reference]	NA	893	68.9 (67.4-70.5)	1 [Reference]	NA
Nonphysician	6672	99.8 (99.7-99.8)	1.00 (1.00-1.01)	.32	320	75.5 (71.1-79.8)	1.10 (1.01-1.19)	.03

Abbreviation: NA, not applicable.

^a^ Regression analysis was conducted for the full-time work indicator by spouse’s occupation (physicians or nonphysician), adjusting for both spouses’ ages (25-29, 30-34, 35-39, 40-44, and 45-50 years), youngest child’s age (no children and 0-3, 4-6, and ≥7 years), number of children (0, 1, 2, and ≥3), urbanicity of residence, prefecture, and year indicators. Multivariable modified Poisson regression models with standard errors clustered at the prefecture level were used. Census-provided weights were used. Adjusted full-time work rates were calculated according to spouse’s occupation using marginal standardization. *P* values < .05 were interpreted as statistically significant.

^b^ The other occupation group consisted of individuals with non–high-income occupations and those who were unemployed.

^c^ Interactions between spouse’s occupation and age of youngest child were tested using the Wald test (*P* = .83 and *P* < .001 for male and female physicians, respectively).

For female physicians without children, adjusted full-time work rates of those married to physicians or nonphysicians were similar (86.0% vs 87.4%; *P* = .52). However, among female physicians with a youngest child aged 0 to 3 years, adjusted full-time work rates were lower for women married to physicians vs nonphysicians (53.9% vs 66.1%; *P* < .001). Similar differences remained when the youngest child was aged 4 to 6 years or 7 years or older.

## Discussion

This cross-sectional study in Japan found that having a physician spouse was associated with lower full-time work rates among female physicians with children. This outcome may be associated with physician spouses’ higher income levels compared with nonphysician spouses. Alternatively, female physicians engaged or married to physicians may choose specialties considered to be parenting friendly and part-time work. Study limitations included unmeasured specialties and individual income and use of cross-sectional data. Generalizability to other countries is also unknown. Nevertheless, these findings aligned with prior US-based studies,^3,4^ despite US-Japan differences. These include a lower ratio of female to male physicians^3^ and more generous public childcare support in Japan,^6^ which legally grants 1-year paid parental leave and provides government-subsidized access to babysitters, childcare facilities, and after-school programs regardless of income level. Nearly 90% of preschoolers (ages 3-5 years) attend government-funded childcare facilities at subsidized rates.^6^

Full-time physician work often involves on-call responsibilities and long overtime hours. This may be associated with pressure on child-rearing female physicians to move to part-time work based on gender norms and domestic expectations. Our findings suggest that this choice for female physicians was accelerated for those married to another physician. Reducing overwork and providing flexible work environments (eg, shift work) may help child-rearing physicians, especially those in dual-physician marriages, continue their careers.

## Notes

Supplement.
eMethods

eReferences
