# Error in Table and Wording

**DOI:** 10.1001/jamanetworkopen.2022.49990

**Published:** 2022-12-14

In the Research Letter titled “Full-time Work Rates of Physicians With Physician Spouses vs Nonphysician Spouses in Japan,”^1^ published November 14, 2022, there was an error in the number and percentage of male physicians with physician spouses who had 0 children listed in Table 1. In addition, “women” was changed to “female physicians” and “men” was changed to “male physicians” to clarify that participants reported sex rather than gender. This article has been corrected.^1^
