# Supplementary material for: Full-time Work Rates of Physicians With Physician Spouses vs Nonphysician Spouses in Japan
Source: JAMA Netw Open. 2022 Nov 14;5(11):e2242143. doi: 10.1001/jamanetworkopen.2022.42143 (PMC9664262; doi:10.1001/jamanetworkopen.2022.42143)
Supplement: Supplement. — eMethods eReferences [file jamanetwopen-e2242143-s001.pdf]

Supplemental Online Content

Miyawaki A. Full-time work rates of physicians with physician spouses vs nonphysician spouses in Japan. *JAMA Netw Open.* 2022;5(11):e2242143. doi:10.1001/jamanetworkopen.2022.42143

eMethods
eReferences

This supplemental material has been provided by the authors to give readers additional information about their work.

© 2022 Miyawaki A et al. *JAMA Network Open.*

eMethods.

Details of the Japan Population Census

The current study is a cross-sectional study using data from the Japan Population Census, a census survey conducted every five years on October 1 via enumerators, mail, or online platforms. This survey is conducted in accordance with the Statistics Act; therefore, all residents of the whole territory of Japan are obliged to respond. The survey collects information on the family structures, demographics, and employment for each household. For example, the 2015 Population Census surveyed household members on the following topics.

For household members:
(1) Name
(2) Sex
(3) Year and month of birth
(4) Relationship to the head of household
(5) Marital status
(6) Nationality
(7) Duration of residency at the current domicile
(8) Place of residence as of 5 years ago
(9) Employment status
(10) Name of establishment and kind of business (Industry)
(11) Kind of work (occupation)
(12) Employment status
(13) Place of work or location of school

For households:
(1) Type of household
(2) Number of household members
(3) Type and tenure of dwelling
(4) Type of building and number of stories

Validity of self-reported occupation

Occupations are self-reported in an open-ended format. Reported occupations were coded by experienced coders according to the *Japanese Standard Occupational Classification*. This classification has long been used in Japanese statistics and previous studies.[1,2] Detailed occupational classifications (e.g., categorizing internists as "physicians" rather than "health care workers") were performed only for the stratified random sample used in the current study, due to manpower constraints. Although physician identity was self-reported, the numbers of working physicians estimated by the Population Census were comparable to those reported in the Survey of Physicians, Dentists and Pharmacists (mandatory physician census conducted by the government).[3] For example, the 2015 Japan Population Census showed 275,252 working physicians versus 311,205 licensed physicians in the 2014 physician census.

Same-sex couples

Same-sex marriage is not legally recognized in Japan. In the Japan Population Census, same-sex couples have been counted in the census as errors or "living with other relatives" rather than as married couples. Since this study focused on married couples, same-sex couples were excluded by definition.

Validity of the outcome (full-time work indicator)

In the current study, the outcome was a full-time work indicator, dichotomized (1="Primarily worked," and 0="Worked part-time besides housework," "Worked part-time while studying," or "On leave") according to the question "How did you work during the week of September 24–30?" This indicator was chosen because it was present across all three censuses. It had 91% sensitivity and 96% specificity for work of ≥ 35 hours/week, according to the 2005 Census (self-reported hours worked were only measured in 2005 and thus would not have been appropriate). These factors assure the validity of this indicator for quantifying full-time work.

Identification of high-income occupations

To identify high-income occupations, we first calculated mean annual income by occupation using the 2017 Employment Status Survey (ESS).[4] The ESS is a nationwide self-administered survey using a stratified random sample conducted by the Japanese government. The ESS provides information on income distribution for all employed individuals and by occupational categories (**eTable 1**) based on the *Japanese Standard Occupational Classification* (i.e., the common categorization method for the Japan Population Census).

© 2022 Miyawaki A et al. *JAMA Network Open.*

In the ESS, annual income for the primary occupation was self-reported in 16 classes: six classes for 0–2.99 million Japanese yen (JPY; 120 JPY=1 US dollar in 2015) in 0.5 million JPY increments, seven classes for 3–9.99 million JPY in a 1 million JPY increment, plus three classes of "10–12.49 million JPY," "12.50–14.99 million JPY," and "15 million JPY or more." The midpoint of the income class was taken, but the last category was top-coded to 15 million JPY. We then calculated the mean annual income for each occupation category i as follows:

$$average\ annual\ income_i = \frac{\sum_{j=1}^{16} M_j \times N_{ij}}{\sum_{j=1}^{16} N_{ij}}$$

Here, M_j is the midpoint of income class j (0.25, 0.75, … 9.5, 11.25, 13.75, or 15 million JPY), and N_{ij} is the number of employees in the income class category j for the occupation category i. Furthermore, unemployed individuals were classified as having zero annual income. Calculated mean annual income by occupation category was as follows:

**Occupation category**	**Mean annual income, million JPY** [a]
Physicians	10.80
Managerial government officials	7.28
Legal workers	7.00
Executives of companies and organizations	7.00
Ship and aircraft operators	6.88
Other administrative and managerial workers	6.81
Researchers	6.62
Management, finance, and insurance professionals	6.61
Railway drivers	6.00
Engineers	5.60
Non-retail sales representatives	5.31
Teachers	5.09
Production-related administrators	4.47
Authors, journalists, editors	4.37
Sales administrators	4.37
Security workers	4.25
Electric construction workers	4.17
Machine maintenance and repair workers	4.10
Sales agents and brokers (e.g., insurance and real estate)	4.07
Stationary plant and construction machinery operators	4.02
General clerical workers	3.92
Machine assembly workers	3.83
Manufacturing-related and quasi-manufacturing workers	3.76
Mine workers	3.73
Health care workers other than physicians	3.72
Machine inspection workers	3.70
Transport and post clerical workers	3.70
Other transport workers	3.67
Product manufacturing and processing workers (metal products)	3.62
Construction and civil engineering workers	3.59
Musicians, stage designers	3.54
Accountancy clerks	3.46
Religious workers	3.35
Fishery workers	3.35
Rubber and plastic product manufacturing workers	3.28
Ceramic, earth, and stone product manufacturing workers	3.28
Beverage and cigarette manufacturing workers	3.27
Motor vehicle drivers	3.26
Printing and bookbinding workers	3.25
Artists, designers, photographers, film operators	3.15
Product inspection workers	2.94
Wooden and paper product manufacturing workers	2.82
Social welfare specialist professionals	2.82

© 2022 Miyawaki A et al. *JAMA Network Open.*

Forestry workers	2.81
Workers not classifiable by occupation	2.64
Product manufacturing and processing workers (except metal products)	2.55
Couriers and postal deliverers	2.51
Other specialist professionals	2.50
Other product manufacturing and processing workers (except metal products)	2.33
Residential facilities, office buildings and other management personnel	2.25
Office appliance operators	2.20
Care service workers	2.14
Agriculture workers	2.12
Food manufacturing workers	2.02
Retail sales workers	2.02
Outdoor service workers	1.95
Healthcare service workers (unlicensed assistive personnel)	1.95
Domestic hygiene service workers	1.94
Spinning, weaving, apparel, and fiber product manufacturing workers	1.82
Food and drink preparatory workers	1.75
Other service workers	1.61
Customer service workers	1.57
Cleaning workers	1.52
Other carrying, cleaning, packaging, and related workers	1.44
Packaging workers	1.24
Domestic support service workers	0.65
Unemployed [b]	0

[a] Calculated from the 2017 Employment Status Survey.
[b] Unemployed individuals were classified as having zero annual income.

In the current study, occupations with an mean annual income of 5 million JPY or more were considered high-income occupations. We applied this threshold because it approximately corresponds to the top quartile of all employees, according to the ESS 2017. Physicians had the highest mean annual income among the occupational categories examined at 10.8 million JPY. Other high-income occupations included managerial government officials, legal workers, executives of companies and organizations, ship and aircraft operators, other administrative and managerial workers, researchers, management, finance, and insurance professionals, railway drivers, engineers, non-retail sales representatives, and teachers.

© 2022 Miyawaki A et al. *JAMA Network Open.*

eReferences

1. Ministry of Health Labour and Welfare Japan. Statistics of Physicians, Dentists and Pharmacists. Accessed September 20, 2022. https://www.mhlw.go.jp/english/database/db-hss/spdp.html
2. Wada K, Kondo N, Gilmour S. Trends in cause specific mortality across occupations in Japanese men of working age during period of economic stagnation, 1980-2005: retrospective cohort study. *BMJ*. 2012;344(7851): e1191. doi:10.1136/bmj.e1191
3. Tanaka H, Nusselder WJ, Bopp M, et al. Mortality inequalities by occupational class among men in Japan, South Korea and eight European countries: a national register-based study, 1990–2015. *J Epidemiol Community Health*. 2019;73(8):750-758. doi:10.1136/jech-2018-211715
4. Statistics Bureau of Japan. Employment Status Survey. Accessed September 20, 2022. https://www.stat.go.jp/english/data/shugyou/index.html
